# Supplementary material for: Maturase K forms a plastidial splicing complex with a neofunctionalized branching enzyme
Source: Nat Commun. 2026 Mar 23;17:4341. doi: 10.1038/s41467-026-70734-3 (PMC13172542; doi:10.1038/s41467-026-70734-3)
Supplement: Supplementary file 1 — Supplementary Information [file 41467_2026_70734_MOESM1_ESM.pdf]

Supplementary Information for:

Maturase K forms a plastidial splicing complex with a neofunctionalized branching enzyme

Yuanyuan Liang[1], Yang Gao[2], Andrea Fontana[1], Melanie Abt[1], Adam Gicgier[1,3], Muriel Gehring[1], Chun Liu[1,4], Mayank Sharma[1], Reimo Zoschke[2], Samuel C. Zeeman[1], and Barbara Pfister[1,*]

[1] Institute of Molecular Plant Biology, ETH Zurich, Auguste-Piccard-Hof 1, 8093 Zurich, Switzerland

[2] Max Planck Institute of Molecular Plant Physiology, Am Mühlenberg 1, 14476 Potsdam-Golm, Germany

[3] Current address: Department of Crop Genetics, John Innes Centre, Norwich, NR4 7UH, UK

[4] Current address: Crop Science Centre, University of Cambridge, Lawrence Weaver Rd, Cambridge, CB3 0LE, UK

*Email: barbara.pfister@biol.ethz.ch

This file contains:

Supplementary Figures 1-16

Supplementary Table 1

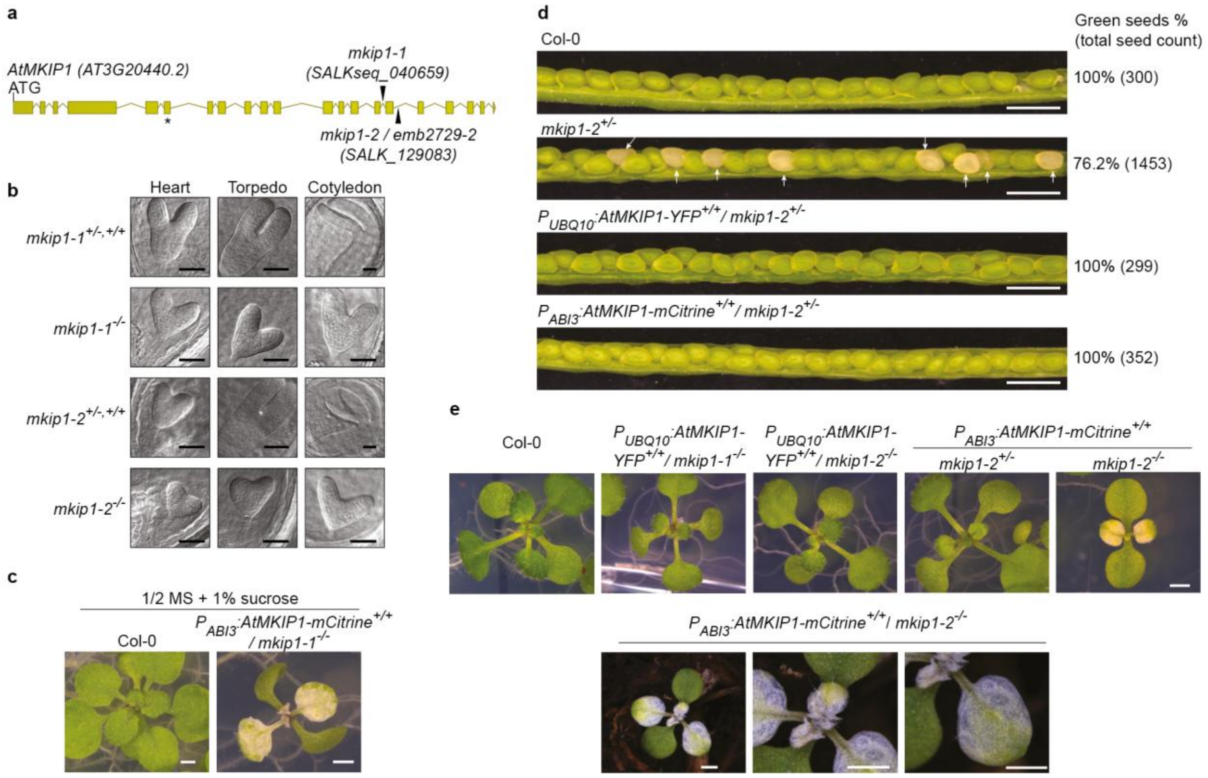

Supplementary Fig. 1. The embryo defective phenotype of Arabidopsis *mkip1* mutants and their complementation by full-length *AtMKIP1* constructs. a Genomic structure of the *AtMKIP1* gene. Exons are represented by green boxes and introns by lines. T-DNA insertion sites of the *mkip1* alleles used in this study are indicated by black triangles. Exon 6 (indicated by an asterisk) is absent in splice form 1 but is present in the primary splice form 2 (AT3G20440.2) used for complementation constructs. **b** Micrographs of wild-type and mutant embryos from siliques of heterozygous *mkip1-1* or *mkip1-2* mother plants at different stages of embryo development. The indicated putative genotypes were inferred from the phenotypes. This experiment was repeated once with similar results. Scale bars: 50 µm. **c** Photographs of 20-day-old seedlings grown on ½-strength MS plates containing 1% (w/v) sucrose. Replicate plants showed similar phenotypes. Scale bars: 1 mm. **d** Opened siliques from mother plants with the indicated genotypes. Seeds with white embryos are indicated by arrows. Percentages indicate the fraction of green seeds of total seeds (number of seeds analyzed in parenthesis). This experiment was repeated once with similar results. Scale bars: 1 mm. **e** Photographs of *mkip1* mutant seedlings rescued by expression of *At*MKIP1 under the constitutive *UBIQUITIN10* (P_{UBQ10}) or seed-specific *ABI3* (P_{ABI3}) promoter. This experiment was repeated once with similar results. At least 10 replicate plants were assessed per line, representative images are shown. Top panel, photographs of 16-day-old seedlings grown on ½-strength MS plates. Bottom panel, light micrographs of a 24-day-old soil-grown *mkip1-2*$^{-/-}$ seedling rescued by the P_{ABI3}:*AtMKIP1-mCitrine* construct. Scale bars: 1 mm.

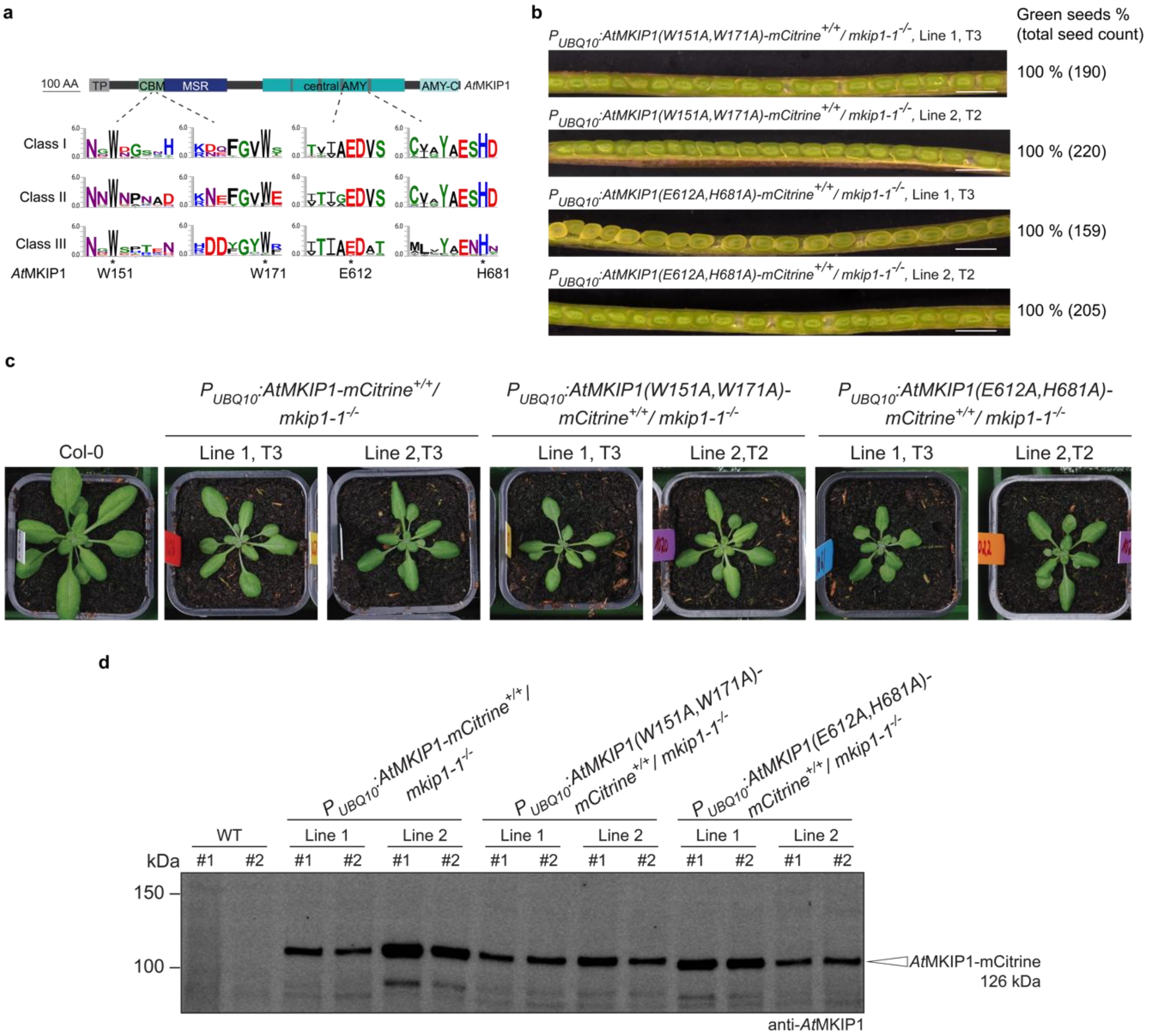

Supplementary Fig. 2. Complementation of the *mkip1-1* mutant phenotype by *At*MKIP1 variants with point mutations in residues conserved among branching enzymes (BEs). a WebLogos showing conserved amino acid residues in the carbohydrate-binding module (CBM) and central catalytic domain (central AMY) based on the sequence alignment of BEs from seed plant species (gymnosperms and angiosperms) used for the phylogenetic tree in **Fig. 2a**. Amino acids in *At*MKIP1 that were mutated to alanines are indicated. **b** Opened siliques from *mkip1-1*$^{+/-}$ mother plants expressing mCitrine-tagged *At*MKIP1 variants. Percentages indicate the proportion of green seeds among total seeds (number of total seeds assessed in parentheses). Two individual lines per construct were evaluated. Scale bars: 1 mm. **c** Photographs of four-week-old wild-type (Col-0) and *mkip1-1*$^{-/-}$ plants complemented with the indicated wild-type or mutant *At*MKIP1-mCitrine constructs. Two independent lines per construct were analyzed. Replicate plants showed similar phenotypes. **d** Immunoblot detection of the *At*MKIP1 versions introduced by the complementation constructs. Two plants per line were analyzed. Total protein was extracted from a four-week-old rosette leaf and loaded on an equal leaf area basis. Native *At*MKIP1 (98 kDa) in Col-0 could not be detected due to insufficient antibody sensitivity.

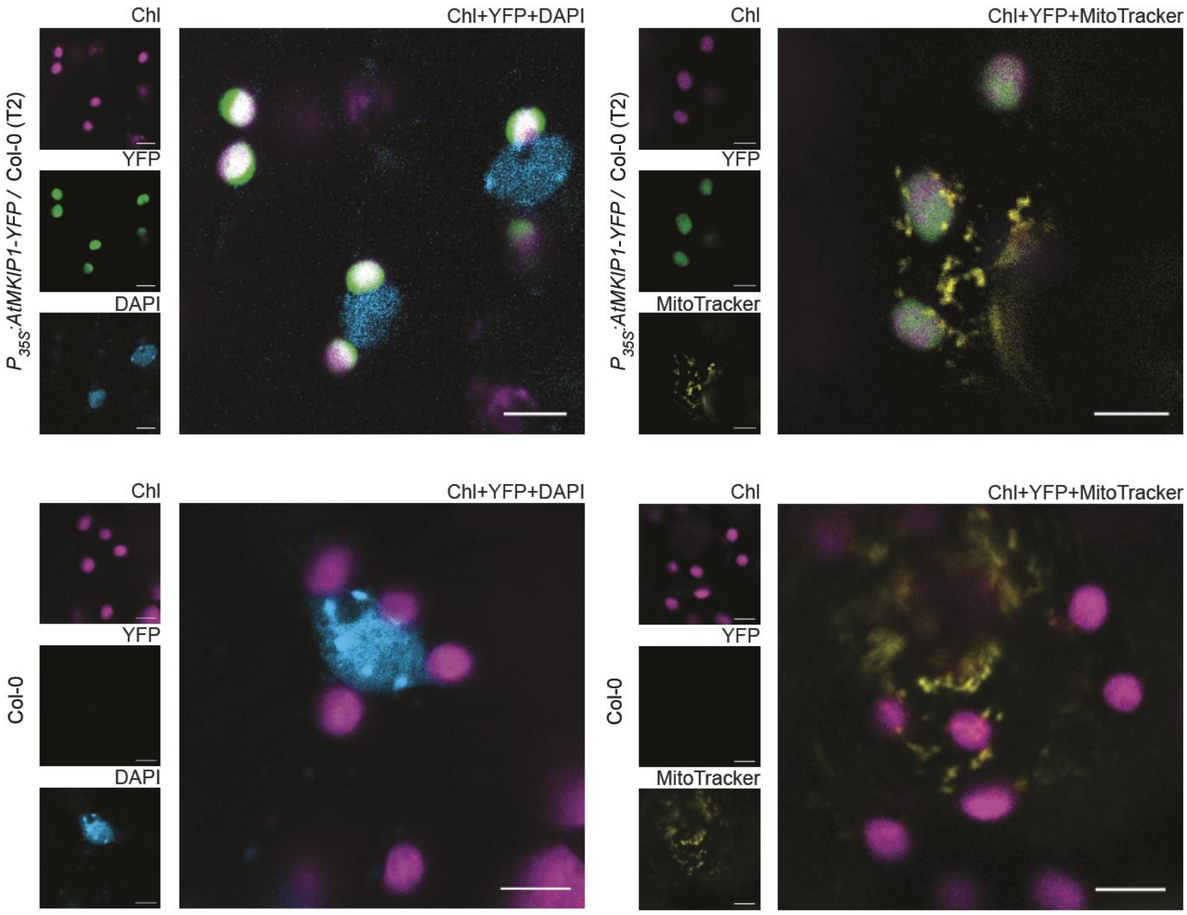

Supplementary Fig. 3. Representative confocal light micrographs of leaf epidermal cells from plants expressing *At*MKIP1-YFP in wild-type (Col-0) Arabidopsis background. Mitochondria (yellow) and nuclei (cyan) were stained with MitoTracker and DAPI, respectively. Chlorophyll (Chl) autofluorescence is false-colored in magenta, and *At*MKIP1-YFP is shown in green. Wild-type control plants (lower panel) were treated and imaged in the same way. *At*MKIP1-YFP signal in mesophyll cells was too weak for visualization. Pixel brightness of individual channels was adjusted uniformly for optimal visualization of signals using ZEN microscopy software. Chloroplast localization of *At*MKIP1-YFP was also observed in another independent line and in a separate experiment involving three independent lines. Scale bars are 5 μm.

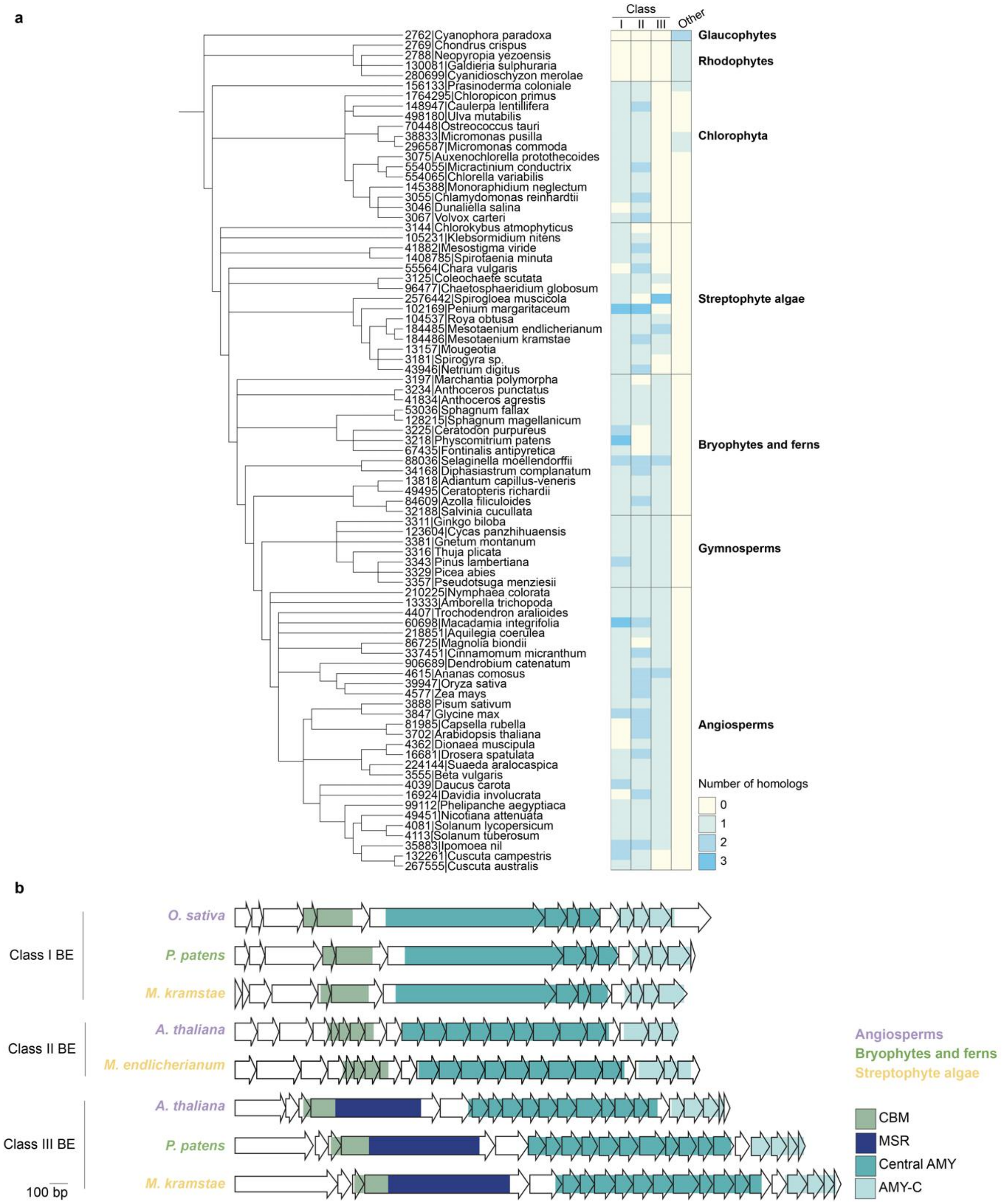

Supplementary Fig. 4. *BE* gene number and structure in *Archaeplastida*. a Phylogenetic tree (left) of the species presented in **Fig. 2a** showing the number of genes from each BE class (right). BE classes are defined according to the phylogeny presented in **Fig. 2a**, with "other" representing the algal BEs that do not fall into classes **I**, **II** or **III**. The species tree follows NCBI taxonomy. **b** Schematic representation of the exon structure of genes belonging to different BE classes from an angiosperm (*Arabidopsis thaliana* or *Oryza sativa*), a moss (*Physcomitrium patens*) and a streptophyte alga (*Mesotaenium kramstae* or *Mesotaenium endlicherianum*). Each arrow indicates an individual exon. Colors indicate the corresponding encoded protein domains.

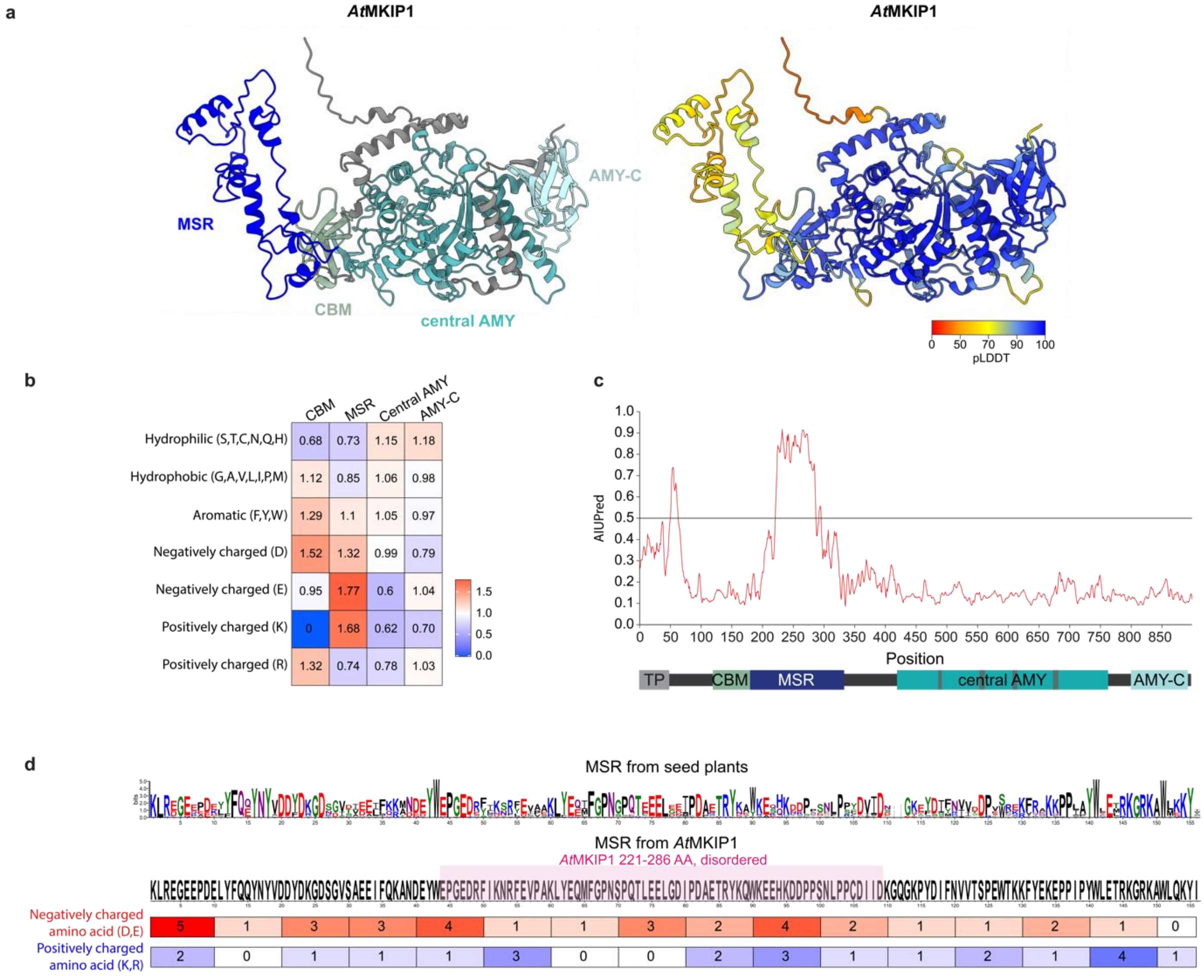

Supplementary Fig. 5. Amino acid composition and structure prediction of the MKIP1-specific region (MSR). a AlphaFold2 prediction of *At*MKIP1 without its chloroplast transit peptide. Structure colored by domain (left panel) or local structural confidence (predicted local distance difference test, pLDDT, where red and blue indicate low and high confidence, respectively; right panel). *At*MKIP1 regions are defined as in **Fig. 2b**. **b** Amino acid composition of *At*MKIP1 regions. The percentage of each amino acid category within each region was normalized to the percentage of that amino acid category in the full-length *At*MKIP1 protein. Amino acids of each category are indicated in brackets. **c** Prediction of disordered regions in *At*MKIP1 by AIUPred. A region with a score >0.5 is generally considered to be disordered. **d** Top panel: WebLogo showing the amino acid conservation in the MSR domain based on the sequence alignment of class III BEs from seed plant species (gymnosperms and angiosperms) used for the phylogenetic tree in **Fig. 2a**. The disordered region predicted by AIUPred is highlighted in pink. Bottom panel: Heatmap of the number of positively and negatively charged amino acids in each ten-amino-acid window within the MSR of *At*MKIP1.

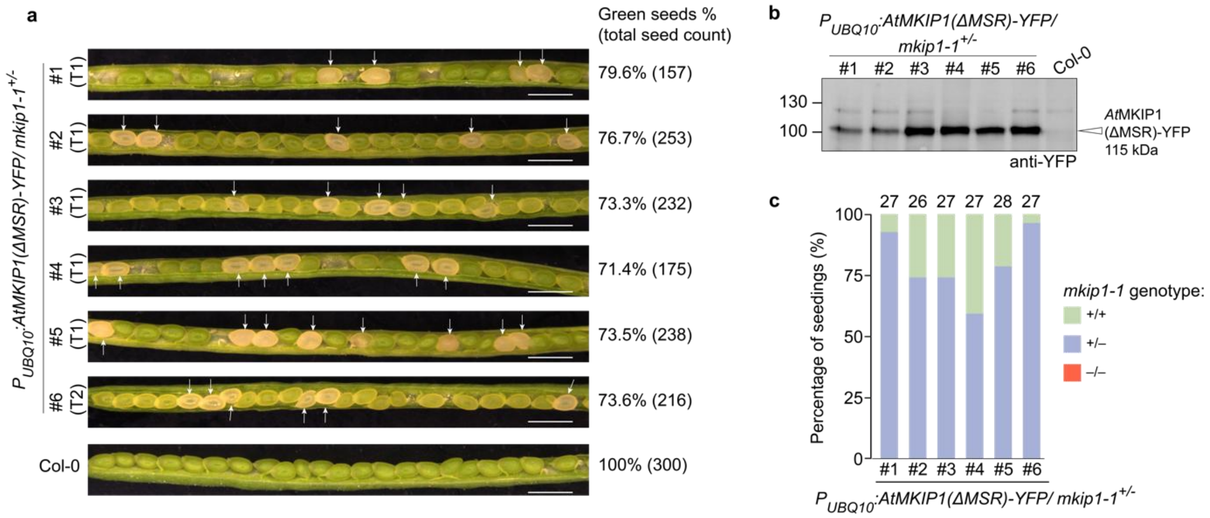

Supplementary Fig. 6. *At*MKIP1 lacking its MKIP1-specific region (MSR) cannot complement the embryo defects of *mkip1-1*. **a** Opened siliques from the wild type (Col-0) and *mkip1-1*$^{+/-}$ transformed with P_{UBQ10}*:AtMKIP1(ΔMSR)-YFP*, in which the MSR (amino acids 178-333) of *At*MKIP1 is deleted. White seeds are indicated by arrows. Percentages indicate the proportion of green seeds in total seeds (number of total seeds assessed in parentheses). Scale bars: 1 mm. **b** Immunoblot analysis of YFP-tagged protein from soluble leaf proteins extracted from 6-week-old rosettes of the mother plants shown in (**a**). Samples were loaded on an equal leaf area basis. **c** Genotyping of the progeny seedlings from the heterozygous *mkip1-1*$^{+/-}$ shown in (**a**). Plants were genotyped for the *mkip1-1* T-DNA insertion. The numbers above the bar indicate the total number of seedlings genotyped in each line. No homozygous *mkip1-1*$^{-/-}$ mutant could be identified for any line.

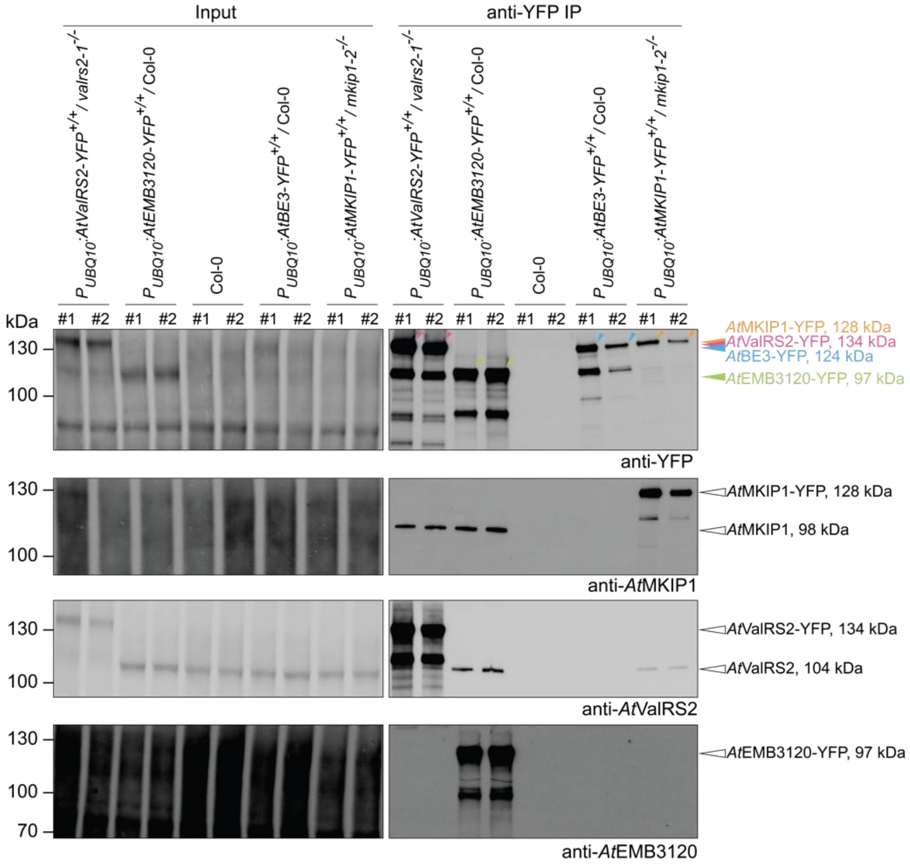

Supplementary Fig. 7. Reciprocal anti-YFP immunoprecipitation (IP) experiments in Arabidopsis using the YFP-tagged interaction partners of *At*MKIP1 as baits. Soluble protein extracted from 10-day-old Arabidopsis seedlings of the indicated genotypes was subjected to anti-YFP IP, using seedlings expressing *At*MKIP1-YFP or *At*BE3-YFP, or no YFP-tagged protein (wild type, Col-0) as controls. Two replicates, each consisting of ~250 mg (fresh weight) of pooled seedlings, were analyzed per line. Soluble extracts were analyzed before IP (Input) and after anti-YFP IP. The predicted molecular weights of the proteins refer to the mature proteoforms without their chloroplast transit peptides. The higher apparent molecular weight of *At*EMB3120-YFP is likely due to its high content of negatively charged residues, which often reduce migration velocity in SDS-PAGE. While *At*EMB3120-YFP co-precipitated endogenous *At*MKIP1, *At*MKIP1-YFP did not detectably co-precipitate endogenous *At*EMB3120 in this set of experiments, presumably due to the lower amounts of input material compared to the experiments shown in **Fig. 3b** and the limited sensitivity of the anti-*At*EMB3120 antibody. Reverse IPs using *At*ValRS2-YFP and *At*EMB3120-YFP as baits were repeated in two other experiments, each of which showed efficient co-precipitation of endogenous *At*MKIP1 by either bait.

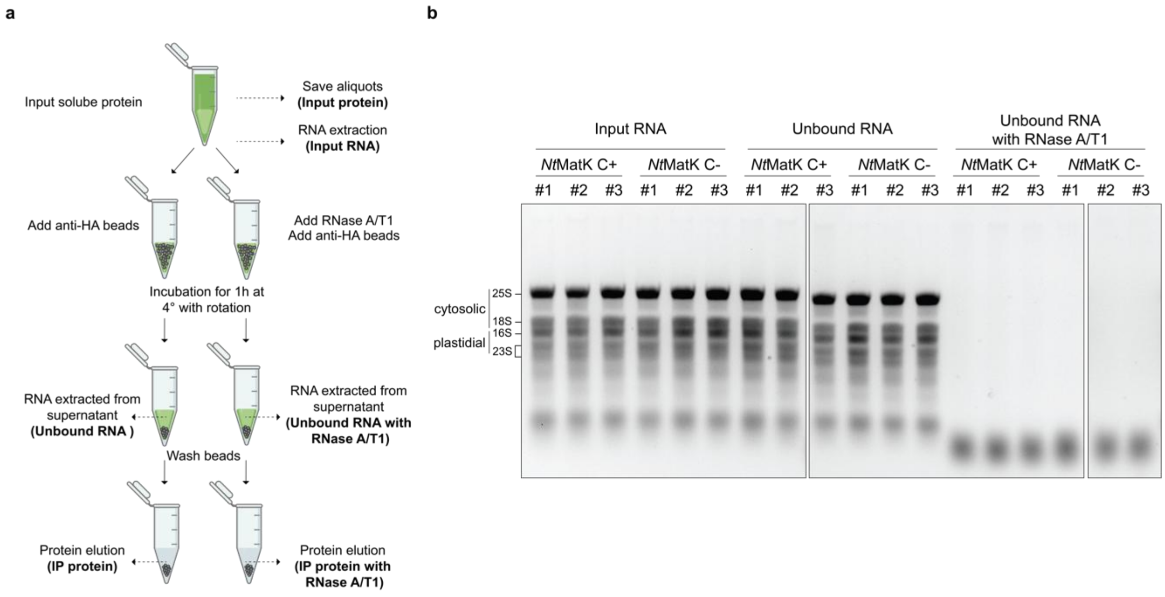

Supplementary Fig. 8. RNA integrity analysis after RNase A/T1 treatment during the tobacco immunoprecipitation (IP) experiment shown in Fig. 3d. a Schematic diagram of the IP procedure with or without RNase A/T1 treatment during the bead incubation step. RNA samples collected for analysis are shown in bold. **b** Gel electrophoresis of RNA isolated during the MatK-HA IP experiment outlined in (**a**). Two µg of RNA were loaded onto a 1.5% agarose gel and visualized by ethidium bromide staining. *Nt*MatK C+ expresses *Nt*MatK with a C-terminal 3xHA tag. *Nt*MatK C- is a control line containing the *aadA* marker gene but no *matK* modification. Three pools of 10-day-old seedlings were analyzed per line.

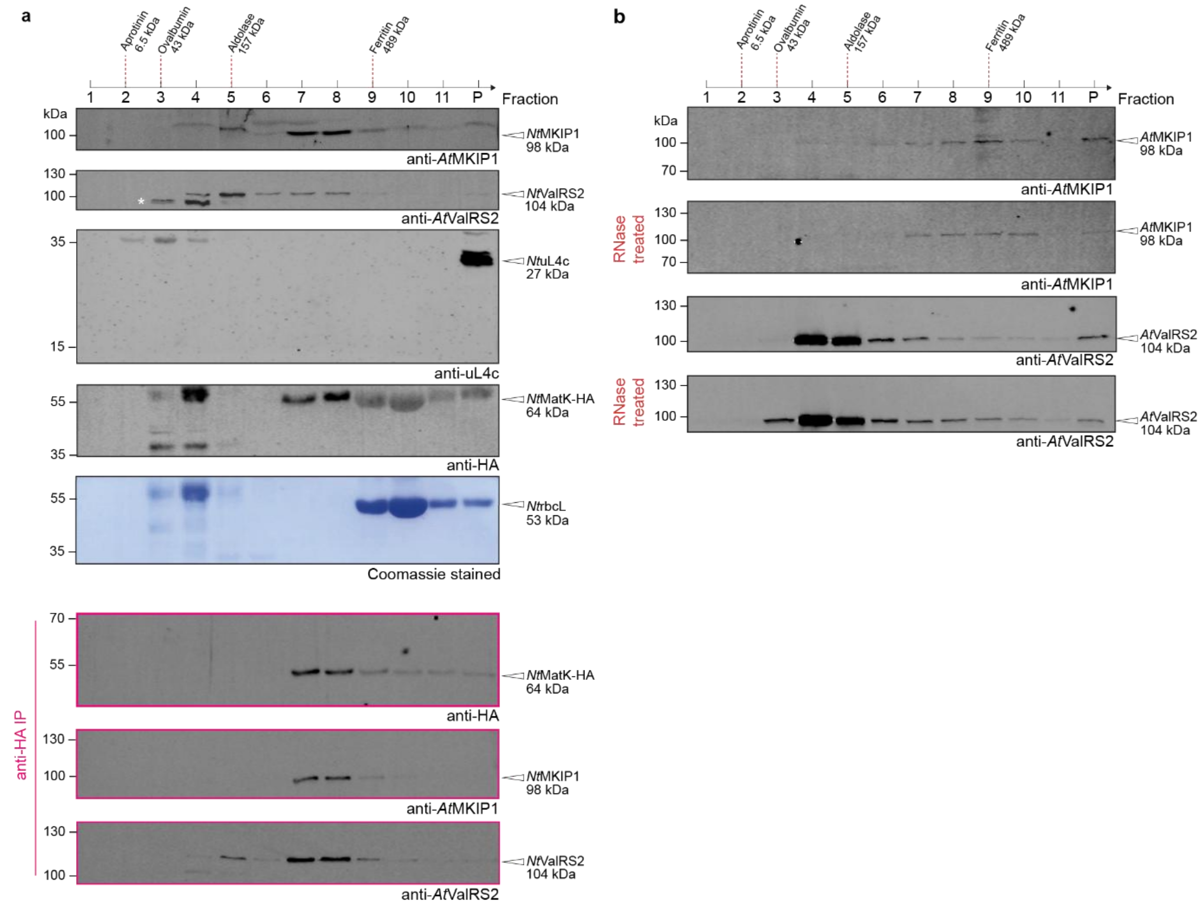

Supplementary Fig. 9. Migration of MKIP1 and its interactors on sucrose gradients. a Sucrose density gradient centrifugation of the same stromal extracts of *Nt*MatK C+ tobacco leaves used for the size-exclusion chromatography shown in **Fig. 4**. Fractions were collected sequentially from top to bottom (fractions 1–11) and assessed by immunoblotting and Coomassie staining. The last fraction constitutes the resuspended pellet (P). The masses and peak fractions of globular protein complexes assessed in parallel are indicated on top. Note that for non-spherical proteins the apparent molecular weights can deviate from those of the markers. The protein indicated by an asterisk may represent a shorter *Nt*ValRS2 version (accession A0A1S4BH67). Since the anti-HA antibody cross-reacted with Rubisco large subunit (*Nt*rbcL), aliquots from the fractions were additionally subjected to anti-HA co-immunoprecipitation (IP), then re-assessed by immunoblotting. *Nt*EMB3120 could not be detected by immunoblotting. **b** Sucrose density centrifugation of stromal extracts of leaves from Col-0 wild type Arabidopsis plants. One half of the extract was additionally treated with RNase A/T1 mixture. Fractions were collected as in (**a**). *At*EMB3120 and *At*MatK could not be detected due to lack of an antibody with sufficient sensitivity.

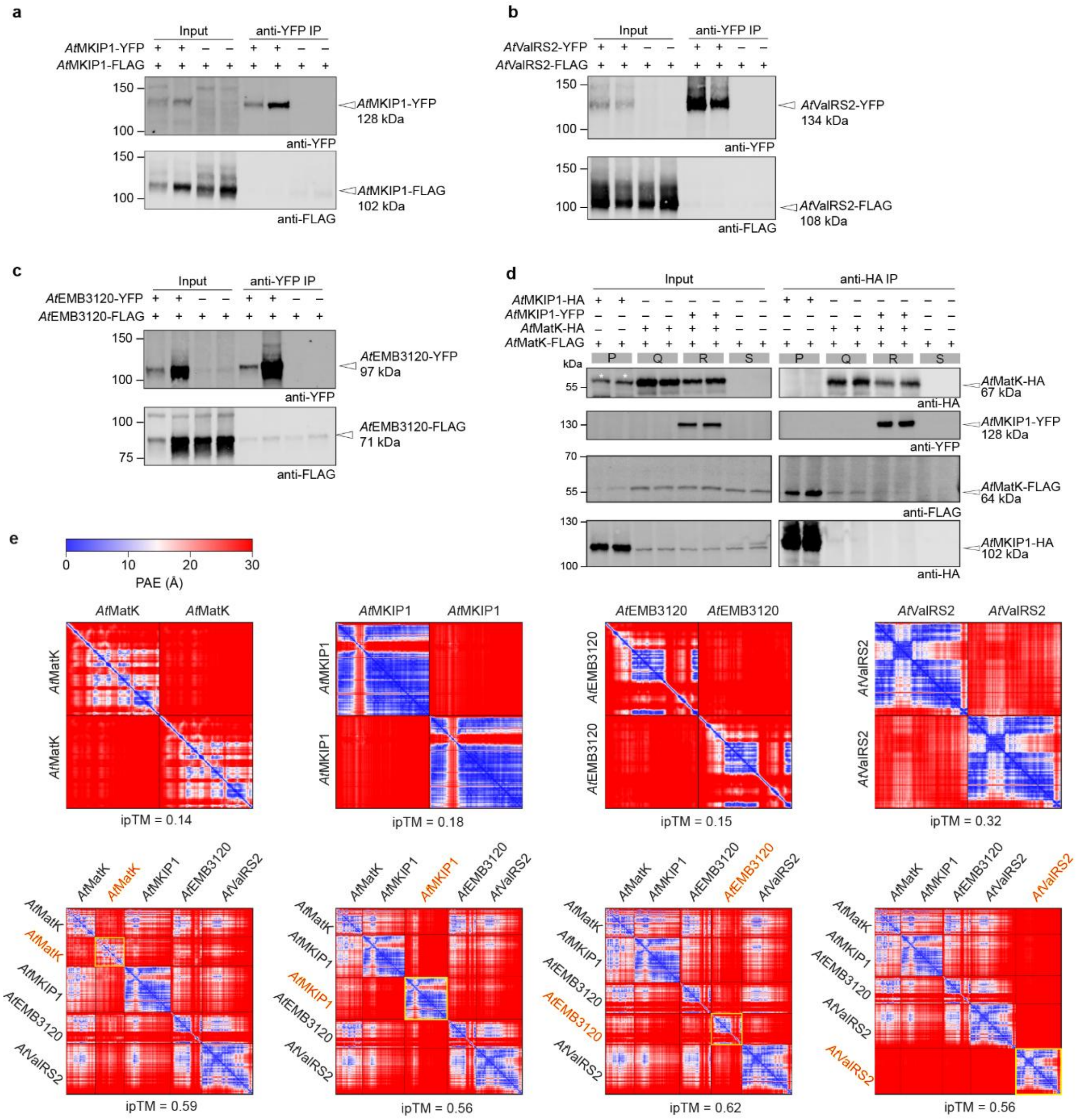

Supplementary Fig. 10. Co-immunoprecipitation (IP) experiments and AlphaFold3 predictions do not support the self-association of *At*MKIP1, *At*ValRS2, *At*EMB3120 and *At*MatK. a-c IP experiments after transient protein expression in *Nicotiana benthamiana* leaves. YFP-tagged proteins were immunoprecipitated using anti-YFP beads (*n*=2 separate plants). The higher apparent molecular weight of *At*EMB3120 is likely due to its high content of negatively charged residues. **d** IP experiments after protein expression in yeast. Soluble protein extracts from yeast strains expressing the corresponding protein (+) or not (-) were analyzed before IP (input) and after anti-HA IP by immunoblotting using the indicated antibodies (*n*=2 replicate cultures). *At*MatK-HA barely co-precipitates *At*MatK-FLAG, indicating that it does not efficiently or stably homo-oligomerize. The bands indicated by asterisks in the anti-HA blot from the input samples probably indicate a degradation product of *At*MKIP1-HA. **e** PAE plots of the top-ranked AlphaFold3 models testing homo-dimerization of the individual subunits (upper panel) or the association of a second subunit copy in the presence of the other complex components (lower panel). The additional copy that was not confidently predicted to associate with the other components is boxed in yellow. ipTM score, interface predicted template modeling score; PAE, predicted aligned error.

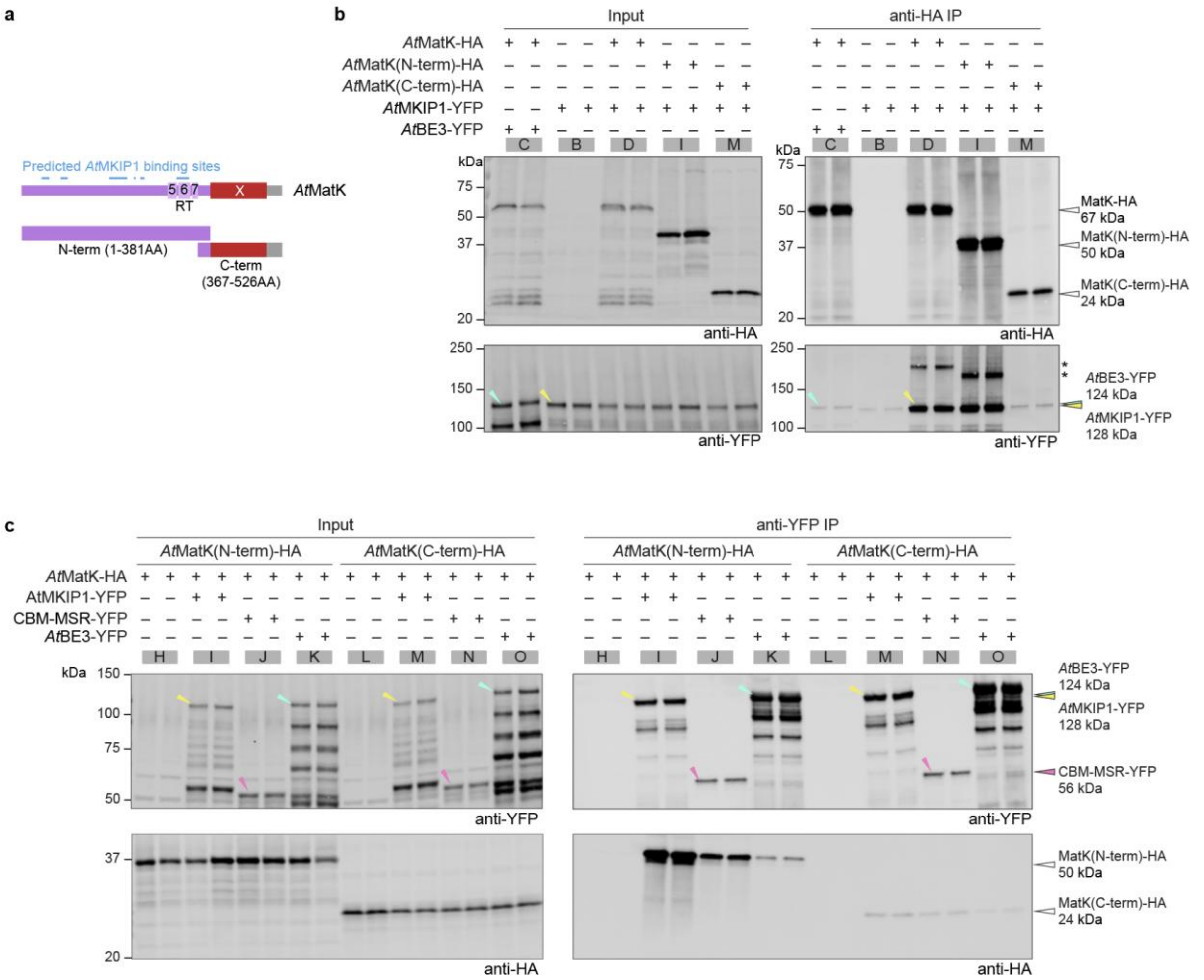

Supplementary Fig. 11. Reciprocal immunoprecipitation (IP) experiments showing the interaction of *At*MatK-HA and *At*MKIP1-YFP in yeast. a Schematics of *At*MatK and the fragments used for testing its interaction with *At*MKIP1. The N-terminal region with its reverse transcriptase (RT) motifs 5-7 (*lilac*) and the X domain (*red*) are indicated. Predicted binding sites (blue lines) are contact sites to *At*MKIP1 that are within 4 Å and have a maximum predicted aligned error (PAE) of 5 Å, according to the AlphaFold3 prediction of the *At*MKIP1-*At*MatK heterodimer. **b** Soluble protein extracts from yeast strains expressing the corresponding protein (+) or not (-) were analyzed before IP (input) and after anti-HA IP by immunoblotting using the indicated antibodies. Two replicate cultures were assessed for each strain. The bands marked with asterisks are of unknown nature but fit the predicted molecular weights of *At*MKIP1-*At*MatK dimers, indicating that a fraction of co-precipitated dimers may be resistant to boiling in SDS. **c** IP experiments performed as in (**b**) but using yeast strains expressing the N- and C-terminal *At*MatK-HA segments shown in (**a**). This figure shows the full IP experiment presented in **Fig. 6g**.

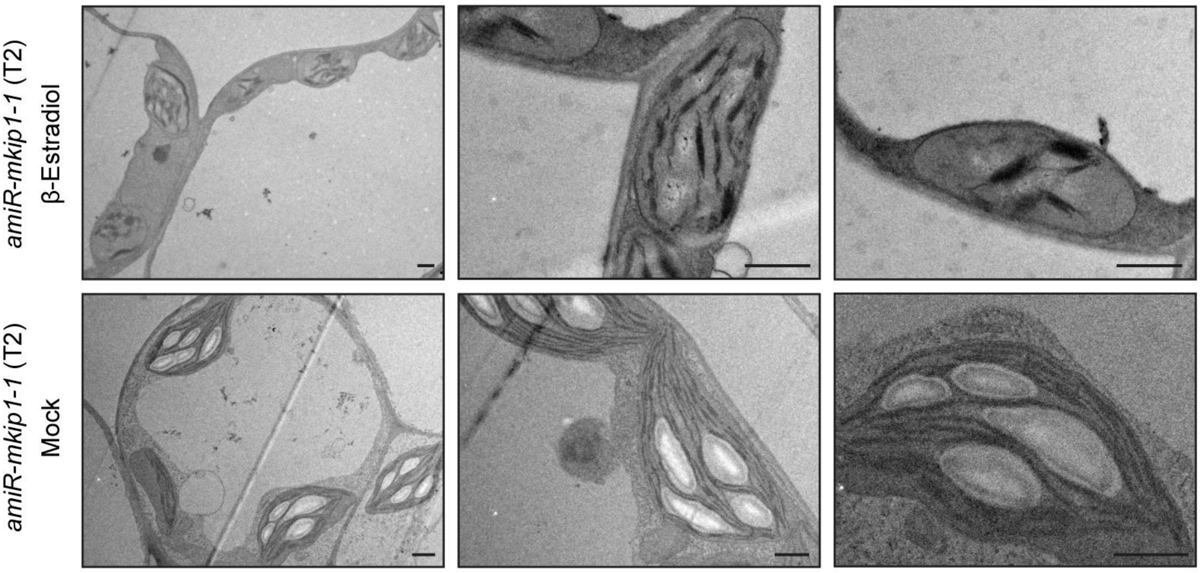

Supplementary Fig. 12. Representative transmission electron micrographs of newly emerged leaves from an *amiR-mkip1-1* plant. Plant treatment with β-estradiol or mock solution and sampling of newly emerged leaves were conducted as described for **Fig. 8**. Plants were derived from an earlier generation than those used in **Figs. 8** and **9**. At least seven plastids were assessed per line, representative images are shown. Scale bars are 1 µm.

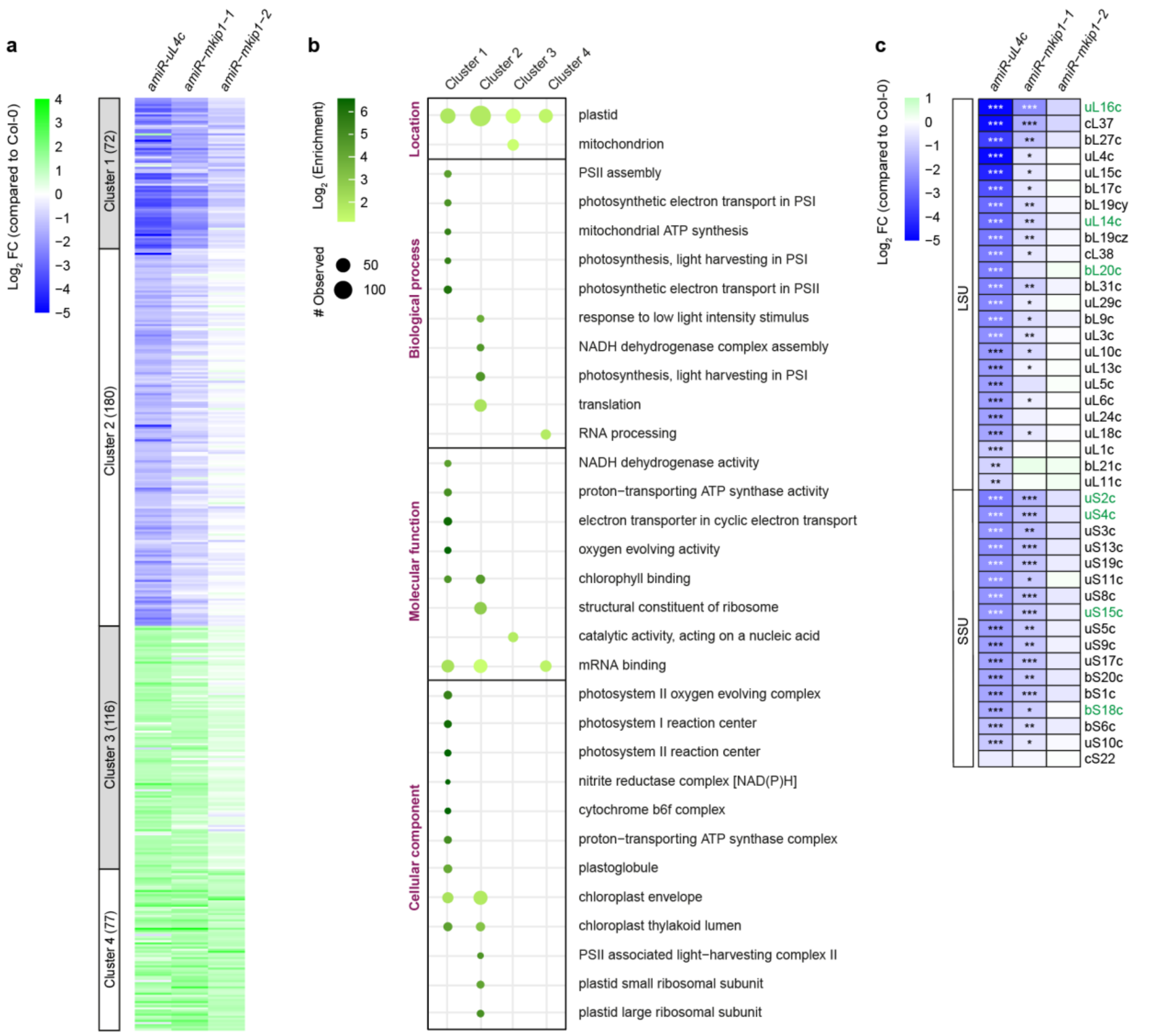

Supplementary Fig. 13. Total proteome analysis after inducing *AtMKIP1* silencing. β-Estradiol treatment, sampling of newly emerged leaves and proteomics analyses were conducted as described for **Fig. 8** (n=4 plants). Col-0, wild type. **a** Heatmap of the $\log_2$ fold changes (FC) of the 445 proteins displaying significant changes compared to Col-0 ($\log_2$ FC ≤ −1 or ≥ 1, adjusted p-value <0.05) in at least one plant line. Green and blue lines indicate proteins that were more or less abundant, respectively, in the indicated line compared to Col-0. Proteins were grouped into four clusters by k-means clustering. Numbers in brackets indicate the number of proteins grouped into each cluster. **b** Subcellular locations and gene ontology (GO) terms enriched in the protein clusters compared to the reference proteome of all quantified proteins. Locations are the consensus locations from SUBA5 database. The GO terms of biological processes, molecular functions and cellular components were retrieved from PANTHER overrepresentation tests. Only locations with $\log_2$ enrichment ≥1 and GO terms with $\log_2$ enrichment ≥1 and adjusted p-value <0.05 are shown. Circle size indicates the observed number of proteins/genes within a location or GO term. Within a GO term family, only the most specific term is shown. Some GO terms were shortened for readability. PS, photosystem. **c** Heatmap of the $\log_2$ fold changes (FC) compared to Col-0 of the ribosomal proteins of the plastidial large (LSU) and small (SSU) ribosomal subunits quantified by proteomics. Proteins in green are encoded in the plastome. The ribosomal proteins uS12cz/uS12cy and uL2cy, which derive from the *rps12* and *rpl2* genes showing splicing defects in *amiR-mkip1*, could not be quantified. Asterisks indicate adjusted p-values (***, adj. p <0.001; **, adj. p <0.01, *, adj. p <0.05). Asterisks are shown in white if $\log_2$ FC ≤ −2. All adjusted p-values are provided in the Source Data.

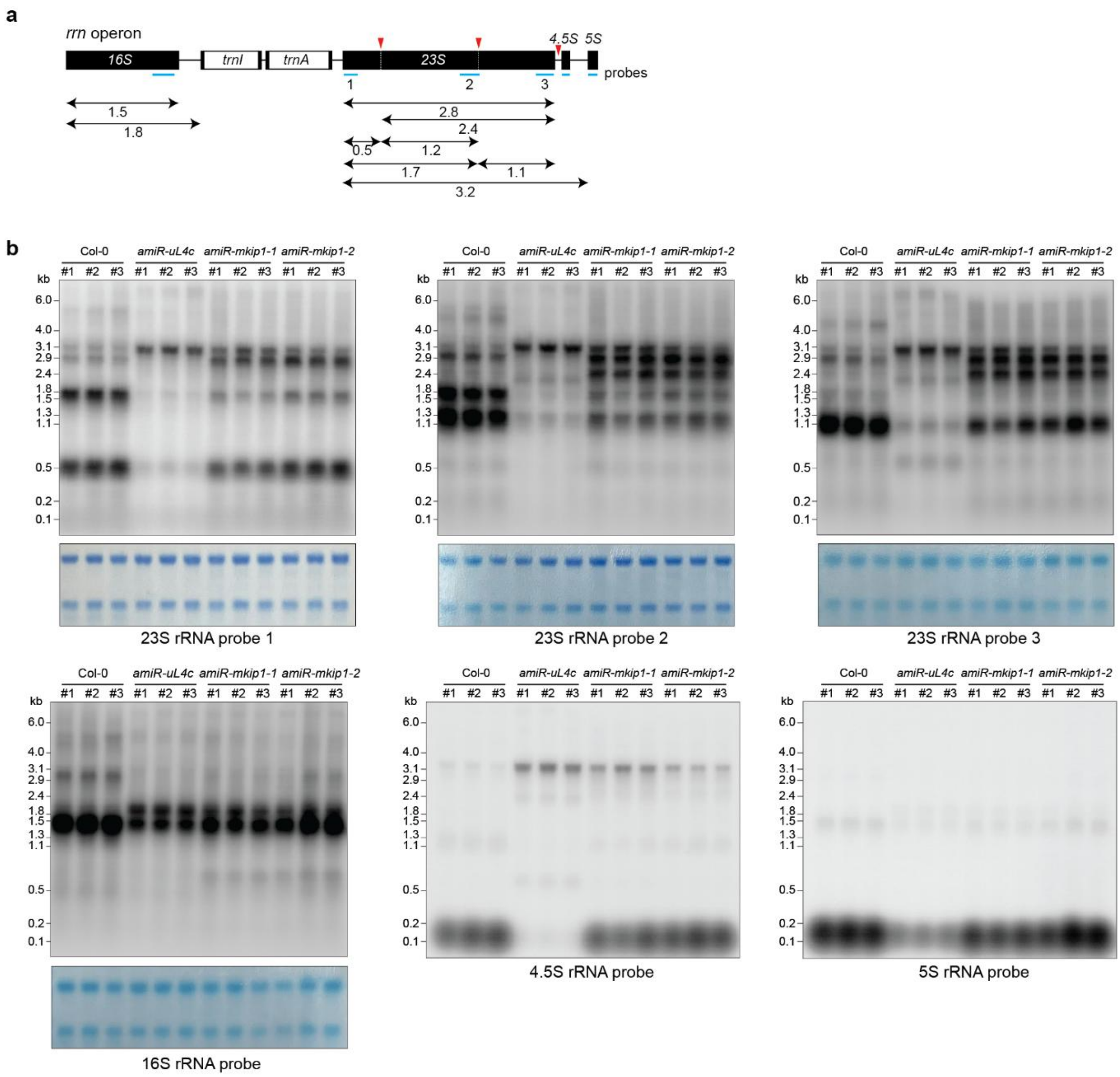

Supplementary Fig. 14. Accumulation of plastidial rRNAs upon silencing of *AtMKIP1* or *AtuL4c*. **a** Scheme of the polycistronic rRNA transcript produced from the *rrn* operon. Subsequent cleavage and trimming steps give rise to the 16S rRNA of the small ribosomal subunit and the 23S, 4.5S and 5S rRNAs of the large ribosomal subunit as well as tRNAs. 23S rRNA is further fragmented into 0.5, 1.2 and 1.1 kb rRNAs by so-called hidden breaks. Red arrows show cleavage steps that probably take place after rRNA incorporation into the large ribosomal subunit. The probes used for northern blotting are indicated in blue and the potentially observed RNA fragments as black arrows, with number indicating their estimated sizes in kb. Exons and introns in the rRNA operon are represented by black and white boxes, respectively. **b** Northern blot analysis of plastidial rRNAs using the probes shown in (**a**). In each lane, 0.5 µg total RNA were loaded. The lower panels show methylene-blue membrane staining (loading control). The membranes used for 23S rRNA blotting (probes 2 and 3) was stripped and re-probed with 4.5S and 5S rRNA probes, respectively. *AtMKIP1* silenced leaves accumulate a 16S rRNA precursor, dicistronic 23S-4.5S rRNA and 23S rRNA intermediates lacking hidden breaks. In treated *amiR-uL4c* plants, rRNA processing appears stronger affected, since fewer mature rRNAs accumulate and 23S rRNA processing is completely blocked already at the level of the 23S-4.5S precursor.

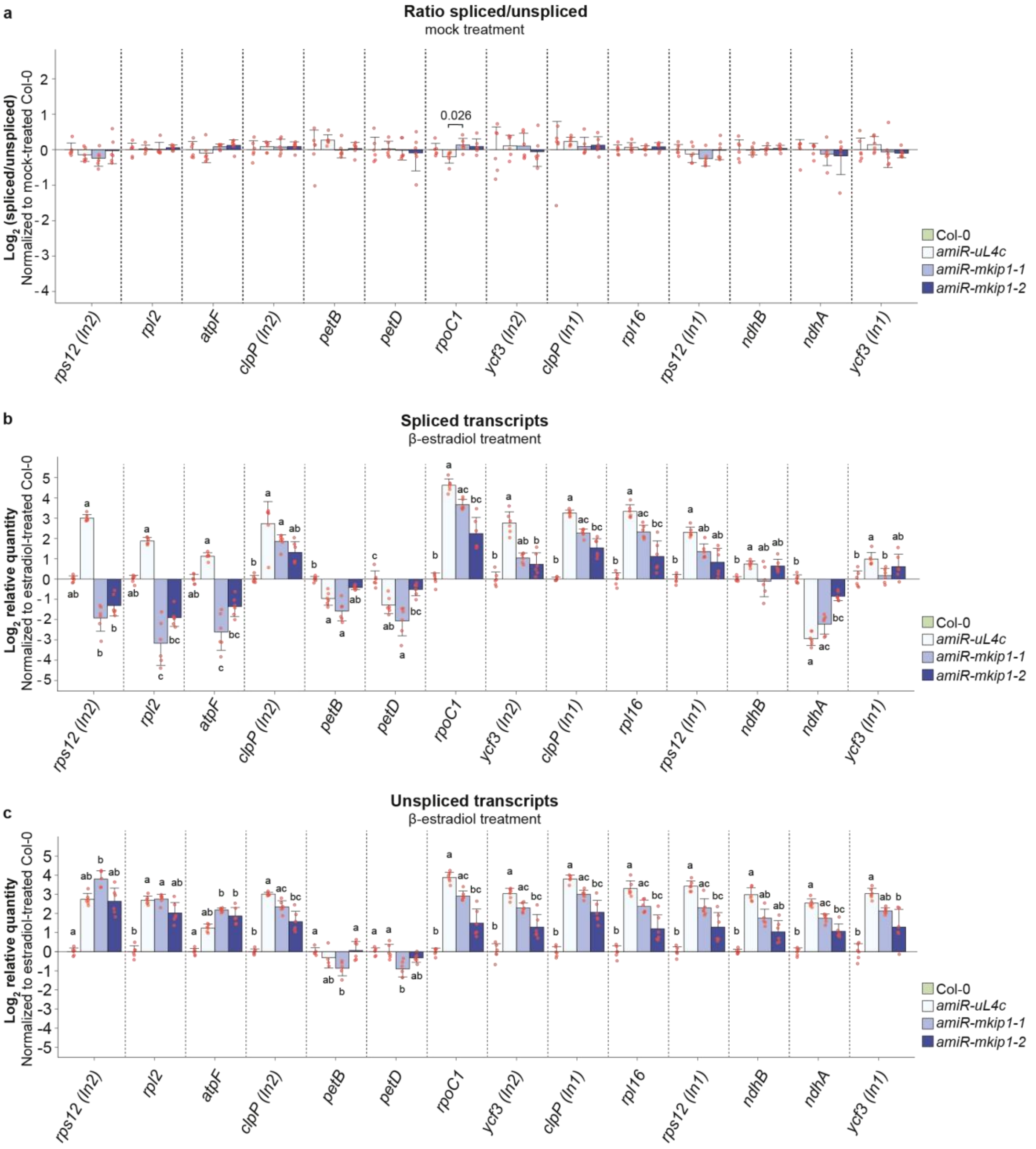

Supplementary Fig. 15. Quantification of spliced and unspliced mRNAs in β-estradiol-treated and mock-treated *amiR* plants by RT-qPCR. a Intron splicing efficiencies in mock-treated plants. Efficiencies were assessed as in **Fig. 9d** but normalized to the mean of mock-treated wild type (Col-0). Shown are means ± s.d. (n=6 plants). Red dots show individual data points. Statistical significance was evaluated using the Kruskal-Wallis test followed by a two-sided Dunn's post hoc multiple comparisons test and Bonferroni p value adjustment. Adjusted p-values <0.05 are provided above the brackets. **b-c** Relative abundances of spliced (**b**) and unspliced (**c**) transcripts in β-estradiol-treated plants. Abundances were first normalized to those of the housekeeping gene *RCE1*, then normalized to the mean of β-estradiol-treated wild type (Col-0). Shown are means ± s.d. (n=6 plants). Red dots show individual data points. Underlying data are the same as in **Fig. 9d-e**. Statistical significance was evaluated as in (**a**). Different letters indicate statistically significant differences (p<0.05) between the means. Adjusted p-values are provided in the Source Data file. An independently repeat of intron splicing analysis in estradiol and mock-treated *amiR-mkip1-1* and estradiol-treated *amiR-uL4c* and Col-0 plants gave similar results.

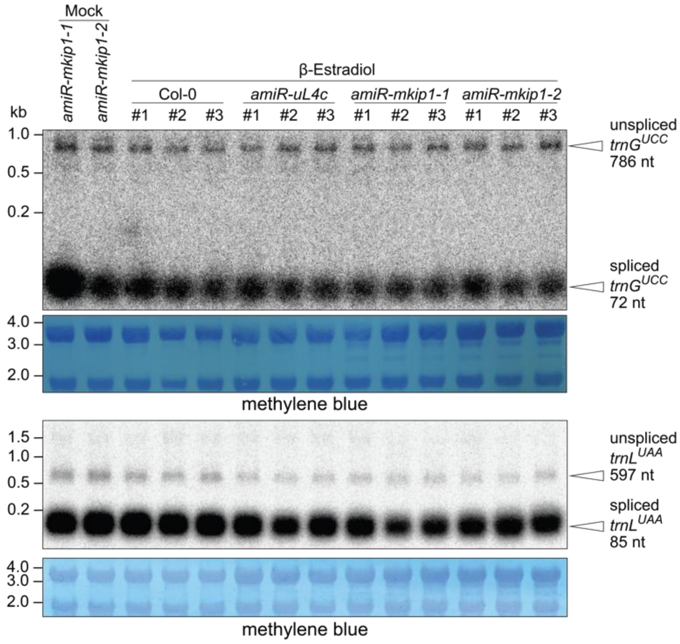

Supplementary Fig. 16. Northern blot analysis of *trnG^UCC* and *trnL^UAA* intron splicing upon induced *AtMKIP1* silencing. β-Estradiol treatment and sampling of newly emerged leaves were conducted as described for **Fig. 8**. Total RNA was separated on denaturing agarose gels, transferred to nylon membranes, and hybridized with probes for *trnG^UCC* exon 2 (top panel) or *trnL^UAA* exon 1 (third panel). Methylene blue is a total RNA stain used as a loading control. Three estradiol-treated plants were analyzed per line. Spliced and unspliced transcripts (with their calculated sizes) are indicated by arrows. Data were used to calculate the splicing efficiencies shown in **Fig. 9**. An independently repeated analysis of intron splicing in estradiol and mock-treated *amiR-mkip1-1* and estradiol-treated *amiR-uL4c* and Col-0 plants gave similar results. Col-0, wild type.

				Quantification (MS1)		Razor spectral counts (MS2)					
						*Nt*MatK C+			*Nt*MatK C-		
**UniProt ID**	**Description**	**cTP**	**Closest Arabidopsis ortholog**	**log_2 FC**	**Adj. *p*-value**	**#1**	**#2**	**#3**	**#1**	**#2**	**#3**
A0A1S4C7U0_TOBAC	*Nt*EMB3120a	yes	*At*EMB3120 (AT3G14900)	8.32	0.0057	157	164	162	0	10	0
A0A140G1P3_TOBAC	*Nt*MatK	n.a.	*At*MatK (ATCG00040)	7.83	0.0007	115	155	137	0	8	0
A0A1S4BH73_TOBAC	*Nt*ValRS2b	yes	*At*ValRS2 (AT5G16715)	7.77	0.0007	196	223	228	0	10	1
A0A1U7Y1B8_NICSY	*Nt*MKIP1a	yes	*At*MKIP1 (AT3G20440)	7.23	0.0003	203	204	239	1	16	1
A0A1S3ZFZ9_TOBAC	*Nt*EMB3120b	yes	*At*EMB3120 (AT3G14900)	7.12	0.0001	9	9	11	0	0	0
A0A1S3Z171_TOBAC	*Nt*MKIP1b-1	yes	*At*MKIP1 (AT3G20440)	6.99	0.0002	5	8	6	0	0	0
A0A1S4A7L4_TOBAC	*Nt*MKIP1b-2	no	*At*MKIP1 (AT3G20440)	5.91	0.0024	49	60	52	0	1	0
A0A1S4AQM5_TOBAC	CRM-domain containing factor	yes	AT3G18390	2.61	0.0003	7	8	6	0	0	0
A0A1S4DJ40_TOBAC	Ribonuclease III domain protein RNC1	yes	*At*RNC1 (AT4G37510)	2.25	0.0003	6	6	6	1	1	1
A0A1S3XJ11_TOBAC	Co-chaperone GrpE family protein	yes	*At*CGE2 (AT1G36390)	1.99	0.0028	5	5	4	0	0	1
A0A1S3XZX4_TOBAC	tRNA ribonuclease (tRNase) Z	yes	*At*TRZ2 (AT2G04530)	1.90	0.2241	8	9	5	0	1	0
A0A1S3XQS5_TOBAC	Nuclear pore complex protein NUP98A-like	n.a.	*At*DRA2 (AT1G10390)	1.80	0.0075	0	4	2	2	1	1
A0A1S4DSL7_TOBAC	Chaperonin-60 alpha	yes	*At*CPNA1 (AT2G28000)	1.77	0.0003	26	26	29	16	17	16
A0A1S4AHG8_TOBAC	Chaperonin-60 beta	yes	*At*CPNB2 (AT3G13470)	1.72	0.0002	37	44	47	22	21	29
A0A1S4BNP6_TOBAC	Chaperonin-60 beta	yes	*At*CPNB2 (AT3G13470)	1.71	0.0007	2	2	3	0	0	1
A0A1S3ZEH5_TOBAC	Ankyrin-repeat protein	yes	*At*STT2 (AT5G66055)	1.68	0.0034	0	2	2	0	0	0
A0A1S4A1Q4_TOBAC	Chaperonin-60 beta	yes	*At*CPNB1 (AT1G55490)	1.52	0.0003	8	8	9	2	2	2
A0A1S4CB32_TOBAC	70 kDa heat shock protein	yes	*At*cpHsc70-2 (AT5G49910)	1.50	0.0002	17	23	21	11	12	12
A0A1S3ZAW0_TOBAC	Heat shock protein 90	yes	*At*HSP90C (AT2G04030)	1.19	0.0022	5	6	6	1	0	1

Supplementary Table 1. List of proteins that are at least two-fold enriched in the *Nt*MatK C+ anti-HA immunoprecipitation presented in Fig. 3e.
Protein quantification for calculation of fold changes (FC) and adjusted (adj.) *p* values base on precursor (MS1) intensities. Statistical significance was assessed within the Amica platform, employing empirical Bayes-moderated two-sided *t*-tests with Benjamini–Hochberg false-discovery rate correction for multiple testing. MS2 razor spectral counts serve as an estimate for peptide abundances. Note that in the MS2 counts each spectrum is ultimately assigned to only one razor (or leading) protein, even if it would match to multiple proteins. For highly similar proteins (such as the "a" and "b" isoforms of *Nt*MKIP1, *Nt*EMB3120 or *Nt*ValRS2), where most peptides can be mapped to both homoeologs, the true distribution of spectral counts assigned to either homoeolog is likely to be more balanced.
The presence of chloroplast transit peptides (cTPs) in the tobacco proteins was predicted by ChloroP. The closest Arabidopsis homolog represents the top BLASTp hit against the Arabidopsis proteome, with the associated locus indicated in parentheses. Arabidopsis orthologs predicted or shown to be plastid-localized (SUBA5 consensus localization) are shown in green.
Since *N. tabacum* is an allotetraploid species resulting from a hybridization between *N. sylvestris* and *N. tomentosiformis*, the isoforms of *Nt*MKIP1, *Nt*ValRS2 and *Nt*EMB3120 were designated as "a" and "b" according to their probable origin (based on amino acid identity) from *N. sylvestris* and *N.*

tomentosiformis, respectively. The origin of the single plastidial *NtMatK* gene is unclear. *Nt*MKIP1a is the full-length MKIP1 from *N. sylvestris*. *Nt*MKIP1a is not annotated in the *N. tabacum* UniProt reference proteome (UP000084051) but shares 100% amino acid identity with *Nt*MKIP1a that we cloned from *N. tabacum* cDNA. We thus consider *Nt*MKIP1a to be a genuine, full-length *Nt*MKIP1.
*Nt*MKIP1b-1 and *Nt*MKIP1b-2 (annotated in the UniProt *N. tabacum* reference proteome) represent N- and C-terminal halves of *Nt*MKIP1 with short extensions. Whether the fragmentation of *Nt*MKIP1b is a sequencing/annotation artifact is currently unclear.
The remaining proteins are likely to be correctly annotated, full-length proteins except for the NUP98A-like nuclear pore complex protein (A0A1S3XQS5_TOBAC), which likely stems from an incomplete sequence resulting in an N-terminal truncation and was hence excluded from the cTP prediction. Only one *Nt*ValRS2 isoform (*Nt*ValRS2b) was quantified because *Nt*ValRS2a and *Nt*ValRS2b are highly similar and the number of peptides uniquely assigned to *Nt*ValRS2a was insufficient for quantification. n.a., not applicable.
