# Supplementary material for: Maturase K forms a plastidial splicing complex with a neofunctionalized branching enzyme
Source: Nat Commun. 2026 Mar 23;17:4341. doi: 10.1038/s41467-026-70734-3 (PMC13172542; doi:10.1038/s41467-026-70734-3)
Supplement: Supplementary file 2 — Description of Additional Supplementary Files [file 41467_2026_70734_MOESM2_ESM.pdf]

Description of Additional Supplementary Files

File Name: Supplementary Data 1

Description: T-DNA insertion plant lines, amiRNA sequences, antibodies, primers for RT-qPCR, yeast vectors, yeast strains and details on the AlphaFold3 predictions used in the present study.
