# Supplementary material for: Maturase K forms a plastidial splicing complex with a neofunctionalized branching enzyme
Source: Nat Commun. 2026 Mar 23;17:4341. doi: 10.1038/s41467-026-70734-3 (PMC13172542; doi:10.1038/s41467-026-70734-3)
Supplement: Supplementary file 4 — Reporting Summary [file 41467_2026_70734_MOESM4_ESM.pdf]

nature portfolio

Corresponding author(s): Barbara Pfister

Last updated by author(s): 18.02.2026

Reporting Summary

Nature Portfolio wishes to improve the reproducibility of the work that we publish. This form provides structure for consistency and transparency in reporting. For further information on Nature Portfolio policies, see our Editorial Policies and the Editorial Policy Checklist.

Statistics

For all statistical analyses, confirm that the following items are present in the figure legend, table legend, main text, or Methods section.

n/a	Confirmed	
☐	☒	The exact sample size (*n*) for each experimental group/condition, given as a discrete number and unit of measurement
☐	☒	A statement on whether measurements were taken from distinct samples or whether the same sample was measured repeatedly
☐	☒	The statistical test(s) used AND whether they are one- or two-sided<br>*Only common tests should be described solely by name; describe more complex techniques in the Methods section.*
☐	☒	A description of all covariates tested
☐	☒	A description of any assumptions or corrections, such as tests of normality and adjustment for multiple comparisons
☐	☒	A full description of the statistical parameters including central tendency (e.g. means) or other basic estimates (e.g. regression coefficient) AND variation (e.g. standard deviation) or associated estimates of uncertainty (e.g. confidence intervals)
☐	☒	For null hypothesis testing, the test statistic (e.g. *F*, *t*, *r*) with confidence intervals, effect sizes, degrees of freedom and *P* value noted<br>*Give P values as exact values whenever suitable.*
☐	☒	For Bayesian analysis, information on the choice of priors and Markov chain Monte Carlo settings
☒	☐	For hierarchical and complex designs, identification of the appropriate level for tests and full reporting of outcomes
☒	☐	Estimates of effect sizes (e.g. Cohen's *d*, Pearson's *r*), indicating how they were calculated

Our web collection on statistics for biologists contains articles on many of the points above.

Software and code

Policy information about availability of computer code

Data collection	Zen (blue edition) was used for microscopy image acquisition. Image Studio (v 6.0) was used for western blot scanning. RT-qPCR data was acquired using LightCycler 480 system (v1.5).<br>No open source or custom code was used.
Data analysis	ImageLab (v6.1 Bio-Rad) was used for analysis of northern blot signals. MAFFT (v7), PhyloBayes MPI (v1.8c), Interactive Tree of life (v6), WebLogo 3, ChloroP, and AIUPred were used for protein sequence analyses. AlphaFold2, AlphaFold3, AlphaBridge and UCSF ChimeraX (v1.10) were used for protein structure analysis and visualization. MaxQuant (v1.6.2.3), the SRMService R package, Philosopher, MSFragger (v3.4 and 4.3), IonQuant (v. 1.7.17) and DIA-NN (v.2.1.0) and the Amica webserver (v3.0.1) were used for mass spectrometry data analysis. Cutadapt and DiffSegR were used for RNA sequence analyses. R (v4.4.1 and v4.5.0) with the R packages data.table (v1.16.2), readxl (v1.4.3), dplyr (v1.1.4), tidyr (v1.3.1), ggplot2 (v3.5.1 and v3.5.2), ggpattern (v1.1.1), FSA (v0.9.5), dunn.test (v1.3.6), rcompanion (v2.4.36), tidyverse (v2.0.0), pheatmap (v.1.0.13) were used for data analyses and plotting. Figures were generated in Adobe Illustrator 2025.<br>All open source codes were described in previous reports. No custom code was used.

For manuscripts utilizing custom algorithms or software that are central to the research but not yet described in published literature, software must be made available to editors and reviewers. We strongly encourage code deposition in a community repository (e.g. GitHub). See the Nature Portfolio guidelines for submitting code & software for further information.

Data

Policy information about availability of data

All manuscripts must include a data availability statement. This statement should provide the following information, where applicable:

- Accession codes, unique identifiers, or web links for publicly available datasets
- A description of any restrictions on data availability
- For clinical datasets or third party data, please ensure that the statement adheres to our policy

All data needed to evaluate the conclusions in this paper are present in the paper and/or its supplementary materials. Source Data are provided with this paper. The proteomics data are freely available at the ProteomeXchange Consortium via the PRIDE109 partner repository with the identifiers PXD060108 [https://www.ebi.ac.uk/pride/archive/projects/PXD060108] (IP data of AtMKIP1-YFP), PXD060055 [https://www.ebi.ac.uk/pride/archive/projects/PXD060055] (IP data of NtMatK-HA), PXD069946 [https://www.ebi.ac.uk/pride/archive/projects/PXD069946] (MS/MS analysis of SEC fractions), and PXD067382 [https://www.ebi.ac.uk/pride/archive/projects/PXD067382] (Proteomics after silencing of AtMKIP1 or AtuL4c). The RNA sequencing data of RNAs bound to AtMKIP1-YFP are publicly available at NCBI GEO110 with the accession number GSE284378 [https://www.ncbi.nlm.nih.gov/geo/query/acc.cgi?acc=GSE284378]. Microscopy images are freely available at the ETH Research Collection [https://doi.org/10.3929/ethz-c-000795755].

Research involving human participants, their data, or biological material

Policy information about studies with human participants or human data. See also policy information about sex, gender (identity/presentation), and sexual orientation and race, ethnicity and racism.

Reporting on sex and gender	We did not conduct any research involving human participants, their data, or biological material in this study.
Reporting on race, ethnicity, or other socially relevant groupings	We did not conduct any research involving human participants, their data, or biological material in this study.
Population characteristics	We did not conduct any research involving human participants, their data, or biological material in this study.
Recruitment	We did not conduct any research involving human participants, their data, or biological material in this study.
Ethics oversight	We did not conduct any research involving human participants, their data, or biological material in this study.

Note that full information on the approval of the study protocol must also be provided in the manuscript.

Field-specific reporting

Please select the one below that is the best fit for your research. If you are not sure, read the appropriate sections before making your selection.

☒ Life sciences ☐ Behavioural & social sciences ☐ Ecological, evolutionary & environmental sciences

For a reference copy of the document with all sections, see nature.com/documents/nr-reporting-summary-flat.pdf

Life sciences study design

All studies must disclose on these points even when the disclosure is negative.

Sample size	Sample sizes were chosen according to standards for experimental plant molecular biology and deemed sufficient to establish the extent of variability and reproducibilty. Most of the techniques used in the present study are of low through-put (e.g. immunoprecipiation, northern blotting). Where feasible, higher number of replicates were used (e.g. several hundreds of seeds were assessed for aborted seed quantification).
Data exclusions	One replicate of the quantification of splicing efficiency of the trnI intron by northern blotting was excluded from the quantification, since it showed non-specific contamination in the area to be quantified. This exlusion is clearly stated in the Figure legend and the contamination is marked with a red asterisk in the northern blot (Figure 9b).<br>We refrained from imputation of the missing values when calculating fold changes of protein abundances in the total proteomics experiments (Fig. 8b, d and Supplementary Fig. 13), as they were rare (<0.15% of the individual protein quantifications) and imputation was deemed artificial. Consequently, non-detected proteins were excluded from the analyses in the respective replicate. This includes the quantification of AtMKIP1 in one replicate of each amiR-mkip1-1 and amiR-mkip1-2, which is described in the figure legend of Fig. 8d.<br>No other data were excluded.
Replication	Most experiments were replicated as a whole (as indicated in the Figure legends) or partially (e.g. we additionally repeatedly tested protein-protein interactions in planta and yeast using partial sets from those shown in the Figures). The findings were replicable in all cases, except for the interaction between AtMKIP1 and AtEMB3120. AtEMB3120-YFP consistently co-precipitated endogenous AtMKIP1 in all experiments. However, while AtMKIP1-YFP did detectably co-precipitate endogenous AtEMB3120 in the set of experiments presented in Fig. 3b, it did not in those presented in Supplementary Fig. 7. Presumably, this is due to the lower amounts of input material compared to the experiments shown in Fig. 3b and the limited sensitivity of the anti-AtEMB3120 antibody. This inconsistency is stated in the legends of Fig. 3b and Supplementary Fig. 7 and in the main text.
Randomization	Plants were grown randomized within the growth chamber. Plants were randomly selected from larger batches for all analyses.

Blinding	Data collection and analysis were not performed blind. In many cases (such as the plants with induced silencing) the identities of the plants were obvious.

Reporting for specific materials, systems and methods

We require information from authors about some types of materials, experimental systems and methods used in many studies. Here, indicate whether each material, system or method listed is relevant to your study. If you are not sure if a list item applies to your research, read the appropriate section before selecting a response.

Materials & experimental systems

n/a	Involved in the study
☐	☒ Antibodies
☒	☐ Eukaryotic cell lines
☒	☐ Palaeontology and archaeology
☐	☒ Animals and other organisms
☒	☐ Clinical data
☒	☐ Dual use research of concern
☐	☒ Plants

Methods

n/a	Involved in the study
☒	☐ ChIP-seq
☒	☐ Flow cytometry
☒	☐ MRI-based neuroimaging

Antibodies

Antibodies used	The antibodies including their dilutions used in this study are listed in Supplementary Data 1.<br>We used the following commercial antibodies: anti-GFP (named anti-YFP in our study; Torrey Pines Biolabs, catalog number TP401), anti-HA (abcam, ChIP Grade; catalog number ab9110), anti-FLAG (Merck, clone M2, affinity isolated antibody, catalog number F1804), anti-PsbA (Agrisera, catalog number AS05 084), anti-RbcL (Agrisera, catalog number AS03 037), anti-RPL4 (named anti-uL4c in our study; Agrisera, catalog number AS15 3076), IRDye 800CW goat anti-rabbit IgG (secondary antibody from LICORbio).<br>We used the following non-commercial antibodies: anti-AtBE3 (from Eurogentec; reported and validated in Pfister, B. et al. (2022), BMC Biol. 20, 1–20.). The anti-AtMKIP1, anti-AtEMB3120 and anti-AtValRS2 antibodies were raised upon request at Eurogentec.
Validation	All commercial antibodies used in this work have been used in several publications since their release. The antibody registration numbers, if available, are as follows: anti-GFP (AB_2313770; http://www.antibodyregistry.org/AB_2313770), anti-HA (AB_307019; https://www.antibodyregistry.org/AB_307019), anti-FLAG (AB_262044; https://www.antibodyregistry.org/AB_262044), anti-PsbA (AB_2172617; https://www.antibodyregistry.org/AB_2172617), anti-RbcL; AB_2175402 (https://www.antibodyregistry.org/AB_2175402). The antibodies from Agrisera have been also validated by the supplier.<br>The non-commercial antibodies were validated by the manufacturer Eurogentec. We also validated them thoroughly using appropriate plant lines (e.g. lines expressing tagged versions of the proteins and/or knockdown lines) and/or yeast strains heterologously expressing the proteins. Data for validation for the are provided within the paper and are described in detailed in Supplementary Data 1.

Animals and other research organisms

Policy information about studies involving animals; ARRIVE guidelines recommended for reporting animal research, and Sex and Gender in Research

Laboratory animals	Our study did not involve laboratory animals.
Wild animals	Our study did not involve animals.
Reporting on sex	Our study did not involve animals.
Field-collected samples	Our study did not involve field-collected samples.
Ethics oversight	Transgenic organisms (Saccharomyces cerevisiae, Escherichia coli, Agrobacterium tumefaciens, Arabidopsis thaliana and Nicotiana tabacum) were handled in accordance with the rules of the Swiss Federal Office for Environment.

Note that full information on the approval of the study protocol must also be provided in the manuscript.

Dual use research of concern

Policy information about dual use research of concern

Hazards

Could the accidental, deliberate or reckless misuse of agents or technologies generated in the work, or the application of information presented in the manuscript, pose a threat to:

No	Yes	
☒	☐	Public health
☒	☐	National security
☒	☐	Crops and/or livestock
☒	☐	Ecosystems
☒	☐	Any other significant area

Experiments of concern

Does the work involve any of these experiments of concern:

No	Yes	
☒	☐	Demonstrate how to render a vaccine ineffective
☒	☐	Confer resistance to therapeutically useful antibiotics or antiviral agents
☒	☐	Enhance the virulence of a pathogen or render a nonpathogen virulent
☒	☐	Increase transmissibility of a pathogen
☒	☐	Alter the host range of a pathogen
☒	☐	Enable evasion of diagnostic/detection modalities
☒	☐	Enable the weaponization of a biological agent or toxin
☒	☐	Any other potentially harmful combination of experiments and agents

Plants

Seed stocks

See "authentication" field below

Novel plant genotypes

See "authentication" field below

Authentication

Seed stocks:
All Arabidopsis thaliana seed stocks were obtained from NASC (The European Arabidopsis Stock Centre) or generated in the course of this work. The following seeds from NASC were used: Col-0 (Columbia) wild type (Name: Col-8; catalogue number/NASC ID: N60000); mkip1-1 (Line identifier: SALKseq_040659; mutant collection: SALK; catalogue number/NASC ID: N540659); mkip1-2 or emb2729-2 (Line identifier: SALK_129083; mutant collection: SALK; catalogue number/NASC ID: N629083); valrs2-1 (Line identifier: SALK_070161; mutant collection: SALK; catalogue number/NASC ID: N570161).
Homoplastomic Nicotiana tabacum (tobacco) lines modified to express NtMatK fused to a C-terminal 3x HA tag (line NtMatK C+) or control lines carrying only the selection marker (NtMatK C-) (described in Zoschke et al. (2010), Proc. Natl. Acad. Sci. USA 107, 3245–3250.) were provided by Christian Schmitz-Linneweber (HU Berlin, Germany).

Novel plant genotypes:
All new plant genotypes of Arabidopsis thaliana were generated by Agrobacterium-mediated transformation using floral dipping (Zhang et al., 2006, Nat. Protoc. 1, 641–646). For complementation analyses, at least two independent lines in the same or in two different mutant backgrounds (mkip1-1 and mkip1-2) were analyzed. Where no complementation was observed (PUBQ10:AtMKIP1 (ΔMSR)-YFP / mkip1-1), 6 independent lines were analyzed. For analyzing the effect of inducible silencing of MKIP1, we used two lines containing distinct amiRNA constructs (targeting distinct regions of the MKIP1 gene), both of which showed similar phenotypes, indicating that off-target effects were absent/negligible.

Authentication:
Seed stocks from NASC were genotyped using the primers described in Supplementary Data 1. All newly generated lines were checked for 1) expression of the transgene in the T1 and additionally in later generations and 2) the number of T-DNA insertions by segregation analyses of the resistance marker in the T2 generation. Only lines with segregation patterns fitting single insertions were used for further analyses. Analyses were generally conducted in the T3 generation, unless otherwise noted in the Figures.
